# The auditory cortex of the bat *Phyllostomus discolor*: Localization and organization of basic response properties

**DOI:** 10.1186/1471-2202-9-65

**Published:** 2008-07-14

**Authors:** Susanne Hoffmann, Uwe Firzlaff, Susanne Radtke-Schuller, Britta Schwellnus, Gerd Schuller

**Affiliations:** 1 Department Biology II, Ludwig-Maximilians-University Munich, Großhaderner Strasse 2, 82152 Planegg-Martinsried, Germany; 2 Institute of Anatomy, Ludwig-Maximilians-University Munich, Pettenkoferstrasse 11, 80336 Munich, Germany

## Abstract

**Background:**

The mammalian auditory cortex can be subdivided into various fields characterized by neurophysiological and neuroarchitectural properties and by connections with different nuclei of the thalamus. Besides the primary auditory cortex, echolocating bats have cortical fields for the processing of temporal and spectral features of the echolocation pulses. This paper reports on location, neuroarchitecture and basic functional organization of the auditory cortex of the microchiropteran bat *Phyllostomus discolor *(family: Phyllostomidae).

**Results:**

The auditory cortical area of *P. discolor *is located at parieto-temporal portions of the neocortex. It covers a rostro-caudal range of about 4800 μm and a medio-lateral distance of about 7000 μm on the flattened cortical surface.

The auditory cortices of ten adult *P. discolor *were electrophysiologically mapped in detail. Responses of 849 units (single neurons and neuronal clusters up to three neurons) to pure tone stimulation were recorded extracellularly. Cortical units were characterized and classified depending on their response properties such as best frequency, auditory threshold, first spike latency, response duration, width and shape of the frequency response area and binaural interactions.

Based on neurophysiological and neuroanatomical criteria, the auditory cortex of *P. discolor *could be subdivided into anterior and posterior ventral fields and anterior and posterior dorsal fields. The representation of response properties within the different auditory cortical fields was analyzed in detail. The two ventral fields were distinguished by their tonotopic organization with opposing frequency gradients. The dorsal cortical fields were not tonotopically organized but contained neurons that were responsive to high frequencies only.

**Conclusion:**

The auditory cortex of *P. discolor *resembles the auditory cortex of other phyllostomid bats in size and basic functional organization. The tonotopically organized posterior ventral field might represent the primary auditory cortex and the tonotopically organized anterior ventral field seems to be similar to the anterior auditory field of other mammals. As most energy of the echolocation pulse of *P. discolor *is contained in the high-frequency range, the non-tonotopically organized high-frequency dorsal region seems to be particularly important for echolocation.

## Background

During the last decade, the bat *P. discolor *has been used increasingly for psychophysical and neurophysiological studies of echolocation [1-4].*  P. discolor *is medium-sized and forages for fruit, nectar, pollen and insects in a neotropical forest habitat. Its vocal emissions are brief (< three milliseconds), broadband multi-harmonic, downward frequency modulated (FM) echolocation pulses with a frequency range of about 40 to 90 kHz. In contrast, the rich repertoire of communication calls used for species-specific social interaction covers the lower frequency range from 11 to 54 kHz [5,6]. The responses of cortical neurons to complex stimuli relevant for echolocation in *P. discolor *have been compared to the behavioral performance of the bat [7,8]. Therefore, it is important to gain knowledge of the detailed organization of the AC with respect to basic response properties.

The auditory cortex (AC) of mammals is composed of distinct fields, which can be characterized by physiological and cytoarchitectural features and their specific thalamo-cortical connections (for review see [9,10]). The functional organization of the AC in bats has been extensively studied physiologically in several species (e.g. *Pteronotus parnellii *[11,12], *Rhinolophus spec*. [13,14], *Eptesicus fuscus *[15,16], for review see [17]). Among the best studied ACs so far are those of the mustached bat *P. parnellii *and the horseshoe bat, *Rhinolophus rouxi*, both belonging to the group of the so-called CF/FM bats whose echolocation pulses consist of a constant frequency (CF) and a frequency modulated (FM) component. As common to all mammals studied so far, their ACs contain a tonotopically organized primary auditory field (AI) with the frequency gradient running from caudal to rostral. However, in both CF/FM bats frequencies of the CF component of the calls are largely overrepresented in AI while frequencies of the FM component are only weakly represented [18]. The AI is surrounded by cortical regions with neurons that show facilitated responses to specific spectral and temporal combinations of the CF and FM parts of the different harmonics of the echolocation pulses.

CF/FM bats are rather specialized echolocators in that they hunt almost exclusively insects on the wings, whereas other bat species display more varied feeding ecology (insects, vertebrates, nectar, fruits) and very commonly use short downward FM echolocation pulses often with several harmonic components. In these bats the functional specialization of the AC is often not so clearly apparent, but still cortical fields can be segregated based on neurophysiological criteria like best frequency (BF; frequency at which threshold is lowest) representation and response threshold [15,19]. In the phyllostomid FM bat *Carollia perspicillata *for example, two dorsal fields containing mainly neurons with BFs in the high-frequency range have been reported in addition to the tonotopically organized fields AI and AAF (anterior auditory field) [20]. In these high-frequency fields some neurons exhibited pulse-echo delay sensitivity as in CF/FM bats [6], but without topographical organization.

Except for a short autoradiographic labeling study [21] the topography of the AC of *P. discolor *has not been studied. In general, the AC of only one other phyllostomid FM bat, *C. perspicillata*, has been investigated, so far [20]. Therefore, the aim of the present study was to investigate neuroanatomical and neurophysiological properties of the AC of *P. discolor *in order to delineate its subdivisions.

## Results

### Auditory responses in the cortex of *Phyllostomus discolor*

**Figure 1:**
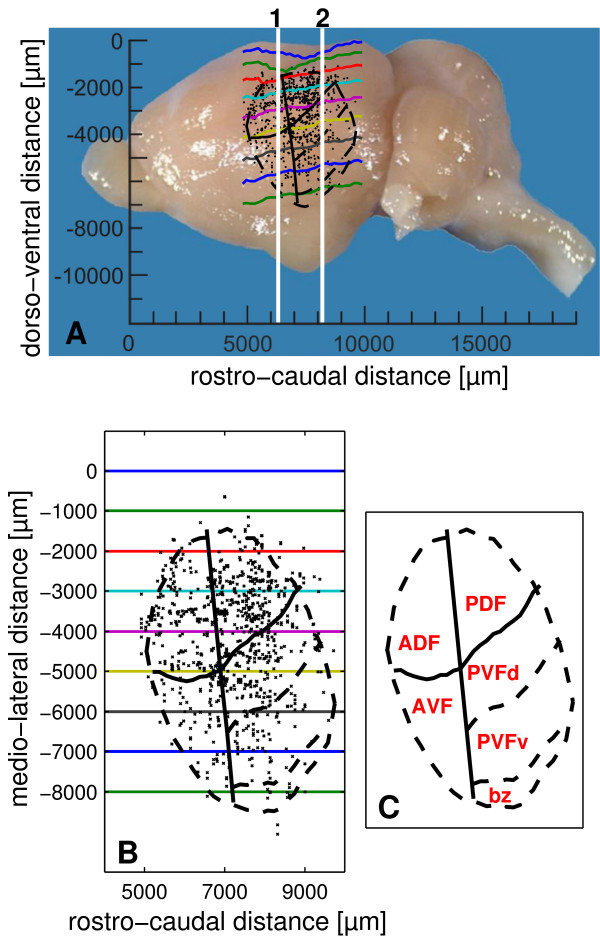
**Recording sites and subfields in the auditory cortex of *Phyllostomus discolor***. A) Lateral view of the *P. discolor *brain. Recording sites of all 849 units are indicated as black dots. Superimposed black outlines are neuroanatomically determined borders. Solid black lines represent reliable borders, whereas stippled black lines represent more variable borders. Rostro-caudal positions of frontal sections shown in Fig. 2 are indicated by the white vertical lines. Colored lines represent equal medio-lateral distances from the midline in 1000 μm steps as shown in the flattened cortical surface projection in 1B. B) Projection of recording sites (black crosses) and neuroanatomical borders (black lines) on an unrolled and flattened cortical surface. Lateral distances on the cortical surface are indicated in 1000 μm steps by corresponding colors as in the side view (1A). The origin used for the flattening process was fixed at 2000 μm lateral from the midline of the brain (upper dark blue line). C) Schematic of the auditory cortical subfields: anterior dorsal field (ADF), posterior dorsal field (PDF), anterior ventral field (AVF) and posterior ventral field (PVF) with dorsal (PVFd), ventral (PVFv) parts and a border zone (bz) reconstructed on the flattened cortical surface. The neuroanatomically determined borders are indicated by black lines.

As shown in Fig. 1A, units responding to acoustic stimuli are found at parieto-temporal portions of the neocortex of *P. discolor*. The distribution of neurophysiological recording sites defines the functional location of the bat's AC. External features roughly delineating the AC are the pseudocentral sulcus [22], which is located at the rostro-dorsal border and the fissura rhinalis, which is located at the ventral border. The auditory cortical area covers a rostro-caudal distance of about 4800 μm and has a dorso-ventral extension of about 5100 μm in the lateral view as shown in Fig. 1A. To obtain a realistic estimate of the cortical surface containing auditory units, the locations of the recorded units were projected on an unrolled and flattened surface projection along the medio-lateral coordinate (Fig. 1B, see Methods). In this projection, the lateral extension of the auditory responsive area is roughly 7000 μm.

### Neuroanatomy

In the area responsive to acoustic stimuli four major fields are recognized based on cyto- and myeloarchitectural features and zinc staining pattern, i.e. the anterior and posterior dorsal fields (ADF and PDF), an anterior ventral field (AVF) and a posterior ventral field (PVF). The PVF maybe further subdivided into a dorsal and a ventral part (PVFd and PVFv) and a border zone (PVFbz), due to minor modifications of the neuroarchitectural characteristics. The topographic position of these fields is depicted in Fig. 1C. Reliable borders of cortical fields are indicated by solid lines. Dashed lines represent the more variable outlines of the AC itself and for PVF possible anatomical subdivisions that are not corroborated by neurophysiological data of the study.

**Figure 2:**
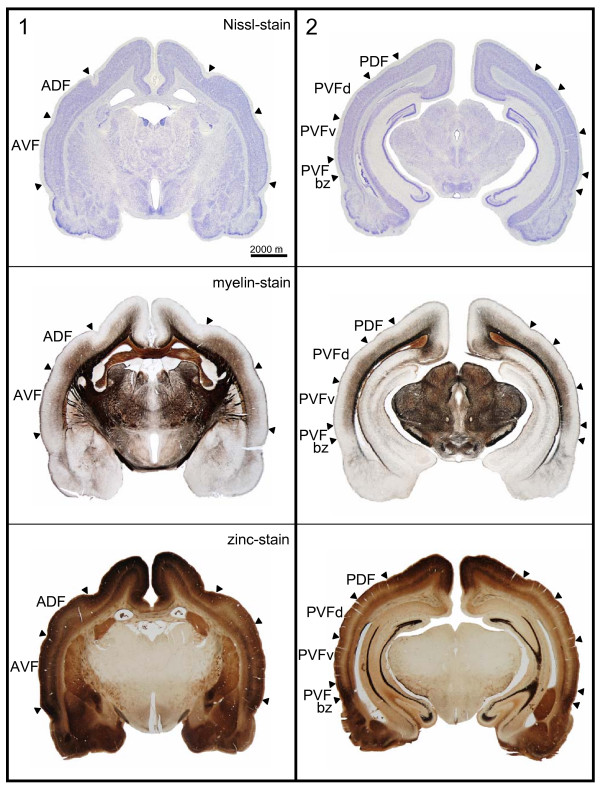
**Frontal sections at two rostro-caudal levels**. The frontal sections are shown for position indicated by the vertical lines in Fig. 1A. Top row: sections (40 μm thick) stained for cells (Nissl); middle row: neighboring sections stained for myelin; bottom row: sections stained for zinc at comparable rostro-caudal level from another series. Scalebar: 2000 μm. Abbreviations as in Fig. 1C.

**Figure 3:**
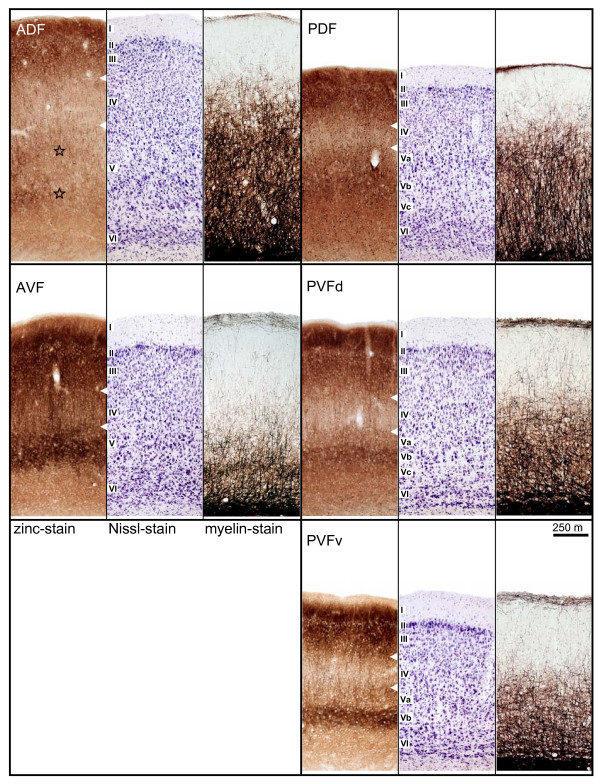
**Cut-outs of frontal sections from the centers of the different cortical fields**. Field names are given in the zinc-stained section and apply to the two neighboring photographs to the right. Indications of layers in the Nissl-stained sections apply to the neighboring left (zinc-stained) and right (myelin-stained) sections, respectively. White arrowheads in the zinc-stained sections indicate the borders of layer IV. Stars in the cut-out of the zinc-stained section of ADF highlight the two components of layer V. The scale bar of 250 μm applies to all cutouts. Abbreviations as in Fig. 1C.

Frontal sections in Fig. 2 give showcase characteristics at two rostro-caudal levels (as indicated in Fig. 1A) to get a general idea of field differences. Total cortical thickness, relative thickness of the different layers, composition of cell types, cell density, content of myelinated fibers and zinc are considered as parameters for the distinction of the different fields. Cut-outs of frontal sections stained for cells (Nissl), myelinated fibers (Gallyas) and for zinc (sulphide/silver histochemical method) from the centers of the different cortical fields are mounted in Fig. 3 for detailed comparison.

Total cortical thickness varies between 1600 μm (ADF) and 1200 μm from dorso-rostral to ventro-caudal locations (PVFv) in the AC. Despite the high density of granular elements in layer III and IV of the dorsal fields, the cortical layers V and VI take more of the total thickness of the cortex. Therefore, they are addressed as parietal cortical fields. The ventral fields are thought to belong to the temporal cortex, as layers III and IV dominate layers V and VI in thickness although granular elements are sparse and the cell density is lower in layers III/IV compared to the dorsal fields.

Dorsal and ventral fields also differ with respect to layer I, which is generally thinner in the dorsal than in the ventral fields. The myelinated horizontal fibers in the superficial part of layer I form a narrow dense band in the dorsal fields, but a broader one in the ventral fields, which is paralleled by a nearly zinc-free stripe in the ventral fields.

The most characteristic features used for delimitation of cortical fields are described in detail as follows. ADF has the greatest cortical width that seems to be caused by a doubled layer V: layer V of the non-auditory cortical area dorsal to the AC seems to continue beneath layer V of ADF (see frontal section stained for zinc in Fig. 2, left column, bottom, and Fig. 3, upper left panel, stars marking the two components of layer V).

In AVF, the rostral beginning coincides with the most caudal part of the claustrocortex without a sharp border. The myelin content in AVF is comparably low, whereas the stain for zinc is generally intense (as is the stain in the rostrally adjacent claustrocortex and the ventrally bordering perirhinal cortex). Even the paler staining band corresponding to layer IV is relatively dark.

The posterior fields are more homogeneous. Cortical width in PDF is smaller than in ADF and characteristic differences to the neighboring PVF are obvious at higher magnification (see Fig. 3). In the zinc stain, layer IV is narrower and more heavily stained in PDF than in PVF, whereas layer V is thicker and of higher staining intensity.

PVF has the most conspicuous wide and pale zinc staining band in layer IV, encompassing deep layer III. Layer V shows three subdivisions in the dorsal part (PVFd), whereas in the ventral part (PVFv) only two subdivisions are recognizable. Layer Vb, the most intense staining part of layer V is strikingly darker in PVFv. In PVFbz a gradual change of the characteristic features from PVF toward the ventrally adjacent perirhinal cortex takes place, e.g. the layered organization fades as well in the cell stain as in the zinc stain, and so do the myelinated fibers.

Layer IV appears as a pale band with the lowest staining intensity in the zinc stain and the staining intensity of layer IV varies characteristically between the fields. It is faint in layer IV of PDF, comparably lighter in ADF, relatively dark in AVF and faintest in PVF. Layer IV (and deep layer III) contains a high number of granular elements in the dorsal fields, whereas the neuronal somata in the ventral fields are larger and the cell density is lower. Layer IV is heavily myelinated in all fields except in the sparsely myelinated AVF.

### Basic neuronal response properties

Because it was not always possible to discriminate the activity of a single neuron, the term 'unit' will be used in the following to describe the activity of one neuron to clusters of three neurons recorded at a distinct recording site. Extracellular recordings were derived from a total of 849 units from both hemispheres of ten bats. The number of units recorded in one bat ranged from 18 (16 penetrations) to 201 (92 penetrations) with an average of 85 units (46 penetrations) per bat (Table 1). Thus, on average two units were recorded per penetration. Recording depths of units derived from roughly orthogonal electrode penetrations (bat #6 and #7) were in the range of 320 to 1700 μm from the cortical surface (n = 183; mean: 962 ± 278 μm).

**Table 1:** Number of electrode penetrations and recorded units per bat

	**Bat 1**	**Bat 2**	**Bat 3**	**Bat 4**	**Bat 5**	**Bat 6**	**Bat 7**	**Bat 8**	**Bat 9**	**Bat 10**
**# of pen.**	16	24	24	27	75	92	56	47	47	47
**# of units**	18	45	36	63	112	201	88	91	106	89

The first row shows the number of electrode penetrations in the AC of each bat, the total number of recorded units in each of the ten experimental animals is shown in the second row.

#### Best frequency and threshold

**Figure 4:**
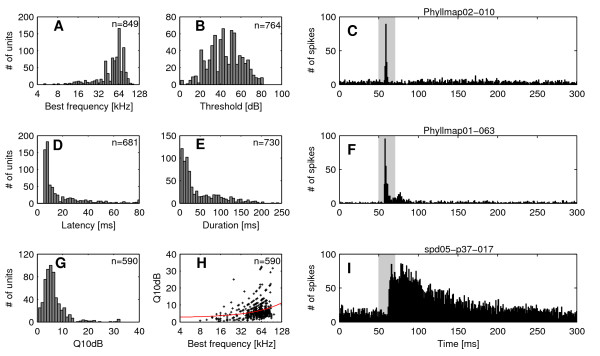
**Distribution of response properties of cortical units and examples of PSTHs for different response types**. The frequency distribution of neuronal response properties evoked by pure tone stimulation is shown for: A) Best frequency, B) Response threshold, D) First spike latency, E) Response duration and G) Q_10dB _values. Peri-stimulus time histograms (PSTHs) show examples of cortical units with different response types: C) phasic response, F) phasic response with a sustained component and I) tonic response. The binwidth of the histograms is 1 ms. The grey bar represents the acoustic stimulus (20 ms pure tone). Panel H) shows the Q_10dB _values as a function of best frequency. The regression line is shown in red.

As shown in Fig. 4A, BFs of units ranged from five to 107 kHz (n = 849; median: 60 kHz; interquartile range: 44 to 67 kHz). Seventy nine percent of the units (674 of 849) had BFs above 40 kHz, i.e. in the range of the dominant harmonics of the echolocation pulse of *P. discolor*.

The response threshold at BF was determined in 764 units and varied between zero and 82 dB SPL. The frequency distribution of thresholds reaches its maximum around 45 dB SPL with roughly symmetric flanks to lower and higher thresholds (mean: 45 ± 16 dB SPL Fig. 4B).

#### Latency and response duration

The first spike latency was measured in 681 cortical units and ranged between five and 127 ms with a bias toward short latencies (Fig. 4D). Median latency of all units was 9 ms (interquartile range: 7 to 20 ms).

Units often exhibited onset responses to pure tones frequently followed by a sustained response component that generally exhibited a considerable amount of variability. Other response types e.g. on-off responses were not observed in the AC of *P. discolor*. The response duration to a 20 ms pure tone was determined in 730 units and ranged from three to 385 ms (median: 25 ms; interquartile range: 14 to 76 ms, Fig. 4E). Short phasic responses with durations below 20 ms (Fig. 4C) were found in 40 % (292 of 730) of units, medium response durations between 20 and 100 ms (Fig. 4F) were found in 44 % (322 of 730) of units and 16 % of units (116 of 730) had response durations longer than 100 ms (Fig. 4I). Thus, in 60 % of units the response duration exceeded the duration of the pure tone stimulus (20 ms).

#### Frequency response areas

Q_10dB _values covered a range between 0.5 and 76 (n = 590; median: 5.4; interquartile range: 3.9 to 7.5). The distribution showed a peak around five (Fig. 4G), indicating that sharp frequency tuning is rare in cortical units of *P. discolor*. The regression line in Fig. 4H shows that Q_10dB _values roughly increased with increasing BFs. Q_30dB _values covered the range between 0.3 and 66 (n = 414; median: 3.1; interquartile range: 2.2 to 4.4). The difference between the medians of the Q_10dB _and Q_30dB _values suggests that in many units the sharpness of frequency tuning was only slightly decreasing with increasing stimulus level.

**Figure 5:**
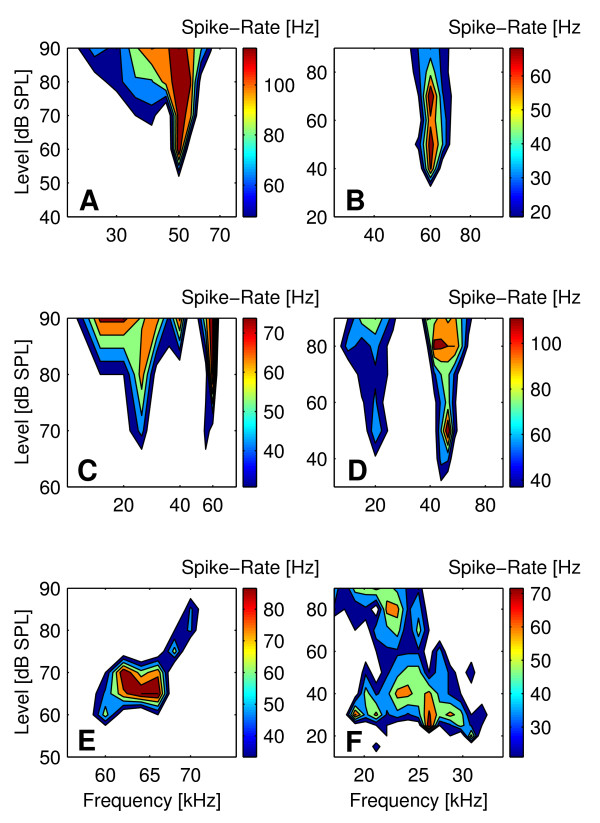
**Examples of the six different FRA types of cortical units**. Examples of the different classes of FRA types are shown for: A) monotonically V-shaped, B) non-monotonically V-shaped, C) monotonically double tuned, D) non-monotonically double tuned, E) circumscribed and F) complex FRAs.

In 745 units, the FRAs could be classified into six different types (Fig. 5). Most units (65 %, 489 of 745) showed a V-shaped FRA with equal share of monotonically (Fig. 5A) and non-monotonically (Fig. 5B) responses. Sixteen percent (120 of 745) of cortical units showed double- or multi-tuned FRAs two thirds (80 of 120) of which displayed monotonic (Fig. 5C) and one third (40 of 120) non-monotonic response behavior (Fig. 5D). In 48 % (58 of 120) of multiple tuned units, threshold minima were roughly harmonically related. In six percent (43 of 745) of units, the FRAs constituted closed areas in the frequency-intensity field with spike rates dropping to zero at all frequencies with increasing stimulus level (Fig. 5E). The remaining units (13 %, 93 of 745) featured complex-shaped FRAs (Fig. 5F).

#### Binaural response properties

In 394 units the binaural response properties were measured and units were classified following their contra versus ipsilateral response characteristics (see Methods). EE responses were measured in 39 % (154 of 394) of units, 32 % (126 of 394) of units were inhibited by the ipsilateral ear (EI) and 25 % (100 of 394) of units showed no reaction to ipsilateral stimulation (E0). Four percent of units (14 of 394), could not be classified in one of the above classes and were thus named as "other".

### Representation of neuronal response properties in cortical fields

The local representation of selected response properties on the cortical surface was visualized using the Voronoi tessellation method (see "Methods"). The tessellation field around the recording site has its limits at halfway distance to the next surrounding recording sites and its color displays the respective response strength of the unit at the center. This forcibly entails large tessellation fields in areas in which recording density is lower or in border regions. As a consequence, there is no proper meaning in the area of single tessellation fields, but rather the clustering or systematic trends of tessellation fields within the AC are important information. Response properties of cortical units were analyzed for the four major subfields. Possible anatomical subdivisions of PVF were not corroborated by the neurophysiological data, and thus, PVF was analyzed as a whole.

#### Representation of best frequency and Q_10dB_

**Figure 6:**
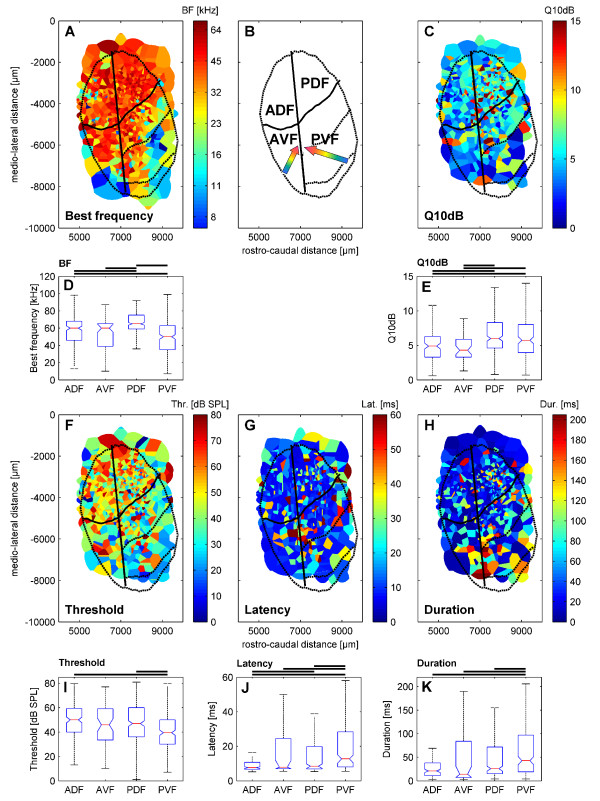
**Spatial representation of response properties on the flattened cortical surface and statistical comparison between the four cortical fields**. Tessellation maps of the spatial representation are shown for: A) Best frequency, C) Q_10dB _value, F) Response threshold, G) First spike latency and H) Response duration. The outlines of AC and AC subfields are superimposed (black lines as in Fig 1). Panel B) shows the topographic position of the AC subfields of *P. discolor *derived from neuroarchitectural characteristics. The arrows indicate BF gradients in the tonotopically organized ventral fields. Statistical analysis of different cortical subfields are shown for: D) Best frequency, E) Q_10dB _value, I) Response threshold, J) First spike latency and K) Response duration. Box plots show the median (red line) and the 25th and 75th percentiles. The 'whiskers' indicate the limits of the remaining percentiles. Outliers (Values >1.5 times the interquartile range) are not shown in the figure. The thick black lines above the plots indicate significant differences (Kruskal-Wallis test, p < 0.05). Abbreviations as in Fig. 1C.

The organization of BFs within the AC of *P. discolor *is shown in Fig. 6A. Units in the dorsal fields had a relatively restricted range of mainly high BFs, which covered frequencies in the range of the dominant harmonics of the echolocation pulse of this species. Most units in the ADF had BFs above 45 kHz, whereas units in the PDF had mainly BFs above 60 kHz. Only at the most rostral positions of the ADF units with low BFs were found. No tonotopic arrangement of BFs was found in the dorsal fields. In contrast, fields in the ventral part of the AC showed a tonotopic organization of BFs. The frequency gradient in AVF developed along the rostro-lateral to caudo-medial axis with low BFs at rostro-lateral positions, whereas in PVF the BFs decreased from rostral to caudal cortical positions. Thus, PVF and AVF shared a common high-frequency border. The rough direction of BF gradients in the ventral fields is schematically shown in Fig. 6B. As shown in Fig. 6D, the median BF (65 kHz) in PDF was significantly higher than the median BFs of the other cortical fields (Kruskal-Wallis test, p < 0.05). The lowest median BF (50 kHz) was found in PVF.

Q_10dB _values of units were also not uniformly distributed on the cortical surface but showed a tendency to increase from anterior to posterior locations (Fig. 6C). In both anterior fields, units showed broader frequency tuning with significantly lower Q_10dB _values (Kruskal-Wallis test, p < 0.05) than found in units in the posterior fields (Fig. 6E).

#### Representation of threshold, latency and response duration

Figure 6F shows the cortical representation of response thresholds at BF within the different fields. Thresholds of units in the dorsal fields were significantly higher than thresholds of units in PVF (Kruskal-Wallis test, p < 0.05) but only slightly higher than those of units in AVF (Fig. 6I). Units in the ADF had highest median response threshold (50 dB SPL), whereas the lowest median response threshold was found in units in the PVF (40 dB SPL).

The distribution of first spike latencies in the AC of *P. discolor *is shown in Fig. 6G. As a general trend, short first spike latencies were preferentially represented in units in the two anterior fields, whereas in units in the posterior fields also long latencies were found. The median latency of PVF (13 ms; interquartile range: 8 to 28 ms) was significantly longer (Kruskal-Wallis test, p < 0.05) than the median latencies of ADF (median: 8 ms; interquartile range: 7 to 11 ms), AVF (median: 8 ms; interquartile range: 7 to 24 ms) and PDF (median: 9 ms; interquartile range: 7 to 20 ms, Fig. 6J).

The representation of response durations within the AC (Fig. 6H) showed the same trend as the representation of first spike latencies: short durations were characteristic for units in the anterior fields, whereas in the posterior fields units with long response durations were found, too. The median response duration of units in ADF (21 ms; interquartile range: 11 to 38 ms) and AVF (14 ms; interquartile range: 7 to 84 ms) were significantly shorter (Kruskal-Wallis test, p < 0.05) than the median response duration of units in PVF (43 ms; interquartile range: 19 to 97 ms) but only slightly shorter than the median response duration of units in PDF (26 ms; interquartile range: 15 to 72 ms). In addition, the median response duration in PDF was significantly shorter (median: 24 ms; interquartile range: 15 to 65 ms, Fig. 6K) compared to PVF.

#### Representation of FRA type and binaural response properties

**Figure 7:**
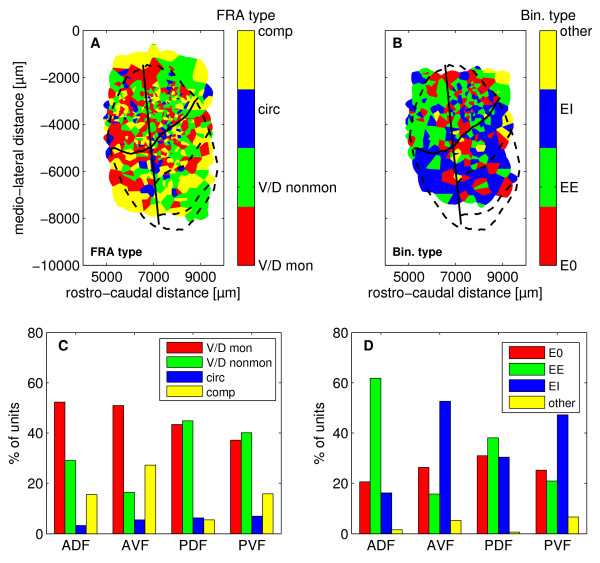
**Spatial representation of FRA types and binaural response types and distribution in different cortical subfields**. Spatial representation A) and distribution in cortical subfields C) of the different FRA types. V/D mon: monotonically V-shaped/double-tuned; V/D nonmon: non-monotonically V-shaped/double-tuned; circ: circumscribed; comp: complex. Spatial representation B) and distribution in cortical subfields D) of the different binaural response types. EI: Excitatory/inhibitory; EE: Excitatory/excitatory; E0: Excitatory/non-responsive. Abbreviation of field names as in Fig. 1C.

The representation of the different FRA-types showed slight differences between anterior and posterior cortical fields (Fig. 7A). Most units of anterior fields had monotonic V-shaped or monotonic double tuned FRAs (ADF: 54 %; AVF: 52 %), whereas non-monotonic V-shaped and non-monotonic double tuned FRAs were mainly found in posterior fields (PDF: 45 %; PVF: 40 %, Fig. 7C). The cortical representation of double tuned FRAs with harmonically related components did not show a specific clustering within certain subfields.

Figure 7B shows the representation of the different binaural response types in the AC of *P. discolor*. The distribution of binaural response types in the dorsal cortical fields was significantly different (Chi-Square test, p < 0.05) compared to the ventral cortical fields (Fig. 7D). Units of the dorsal fields were mainly driven by input from both ears and were classified as EE (ADF: 62 %; PDF: 38 %). In contrast, units of the ventral fields were predominantly characterized by EI input type. In detail, 53 % of units in AVF and 47 % of units in PVF were excited only through the contralateral ear and were inhibited by ipsilateral stimulation.

## Discussion

This study investigated the localization, neuroarchitecture and basic physiological response properties of the auditory cortex of the bat *P. discolor*. The large area of neocortex responsive to acoustic stimulation comprises four anatomically distinguishable fields: a ventral part of the AC with an anterior and a posterior subdivision (AVF and PVF) and a dorsal part of the AC further divided into anterior and posterior subfields, ADF and PDF. The anatomical parcellation of the AC was consistent with the differentiated distribution of basic acoustical response properties of units in the cortical subfields. Location and extension of the AC of *P. discolor *as defined by neurophysiological and neuroarchitectural methods in this study roughly corroborated the findings from an earlier 2-DG study in the same species by Esser [21].

### Comparison with the auditory cortex of other bats

The distribution of BFs in the AC of *P. discolor *(see Fig. 4A) showed an overrepresentation of frequencies in the spectral range of the bat's echolocation pulse (40 to 90 kHz). This overrepresentation might be due to the fact that the dorsal fields are mainly containing units with BFs in the high-frequency range. Thus, a larger amount of cortical surface is devoted to units with high BFs as compared to units with low BFs. In addition, more units were recorded from the dorsal fields and this bias might also be reflected in the distribution of BFs. A similar overrepresentation of BFs in the spectral range of the echolocation pulses has been found in the AC of other microchiropteran bats e.g. *E. fuscus *[23], *Myotis lucifugus *[17] and *C. perspicillata *[20]. In the CF/FM bats *P. parnellii *[18], *R. rouxi *[13] and *R. ferrumequinum *[14] the overrepresentation of a narrow call-specific spectral range of the CF-part is reflected throughout the ascending auditory pathway [24-29] and is based on specializations already implemented on the cochlear level [30-32].

In CF/FM bats, a large part of the AC is involved in processing of specific combinations of the different harmonics in the echolocation pulse and echo. In *P. parnellii *and slightly less distinctive in *R. rouxi*, several fields can be segregated by virtue of selective responses of neurons to different combinations of FM-parts or CF components of call and echo. Neurons are tuned to delays between the components and are topographically ordered [12,17,33]. Such a complex representation of pulse and echo delay has generally not been found in FM-bats so far, although pulse-echo delay sensitive neurons exist in the AC of e.g. *M. lucifugus *[34] and in the dorsal parts of the AC of the phyllostomid bat *C. perspicillata *[6], which is closely related to *P. discolor*. In the present study, only pure tones but no echolocation related stimuli (e.g. FM/FM pairs) were used for acoustic stimulation (see Methods). Consequently, no statements concerning the functional involvement of cortical subfields other than pure tone processing (e.g. delay sensitivity) can be made, so far. Besides that, the organization and parcellation of the AC of *P. discolor *is generally in accordance with that of *C. perspicillata*. The dorsal fields in the AC of *Carollia *were similarly characterized by neurons with BFs in the high-frequency range, and the ventral part of the AC was also composed of two tonotopically organized subfields, which shared a common high-frequency border [20]. In contrast, the AC of other non-phyllostomid FM bats like *M. lucifugus *and *E. fuscus *showed differentiation into subfields to a lesser degree [15,19]. In both species tonotopically organized fields with frequency gradients running in opposite directions were found, but no fields with characteristics similar to the dorsal cortical fields in *P. discolor *were described.

A high percentage (55 %) of units in the AC of *P. discolor *had first spike latencies below 10 ms. As other studies in FM-bats reported mean latencies measured at 10 to 20 dB above threshold around 8 to 10 ms already on the level of the IC [35,36], this seems to be unusually short. However, short latencies in the AC of FM-bats were also reported [37]. In addition, Haplea et al. showed that on the level of the IC latencies in *E. fuscus *still could be as short as about three milliseconds [35]. Although most cortical neurons in *R. rouxi *had latencies in the range between 10 to 15 ms, short latencies below 10 ms were found in a substantial 18 % of neurons [13]. The bias toward short latencies below 10 ms in *P. discolor *might be explained by the fact that a large number of recordings in our study were derived from dorsal regions of the AC where short latencies are predominately represented.

In contrast to findings in other mammals where the AI has been described to have the shortest latencies [38], the lowest median first spike latency in *P. discolor *was found in the ADF, whereas in the PVF, which might be equivalent to the AI, the longest first spike latencies were found. Our findings in *P. discolor *are supported by the fact that in the AC of *R. rouxi *shortest latencies were also found in the dorsal fields [13]. In *R. rouxi*, ventral and dorsal cortical fields are not sequentially connected but rather receive connections from different parts of the auditory thalamus via different projection pathways [39-41]. In *P. discolor*, a similar connection pattern could explain the shorter latencies in the dorsal fields compared to the ventral fields. However, this hypothesis needs to be proved by future tracer studies in this species.

### Influence of anesthesia and multi unit recordings on response patterns

A large proportion of units in the *P. discolor *AC showed onset-type responses. It is known that the response pattern of cortical neurons is influenced by anesthetics. For example, the use of barbiturates like pentobarbital that enhances GABA_A_-mediated inhibition biased the distribution of response types toward short onset responses in the rat auditory cortex [42]. The drug Midazolam used in our study, like other benzodiazepines, also enhances action of GABA on GABA_A _receptors. Thus, the high abundance of phasic onset-type responses in our study could be a consequence of the use of Midazolam. However, a high percentage of onset-type response patterns (68 %) were also found in neurons in the AC of awake *R. rouxi *[13] and awake mustached bats [43], indicating that even in the un-anesthetized bat phasic responses are common. This minimizes the potential effect of anesthetics on response types of cortical units in the present study. Another point that could have influenced the response pattern is that recordings in our study were not always derived from single units (see Methods). Seshagiri and Delgutte [44] showed that adjacent neurons recorded simultaneously with tetrodes in the inferior colliculus of cats could differ significantly in their temporal discharge pattern although BFs and threshold were highly correlated. Therefore, blurring of response pattern is possible in some cases in multi unit recordings, but markedly dependent on the number of contributing neurons with dissimilar response types. As multi unit recordings in this study were generally derived from only up to three neurons (see Methods), and most units showed phasic responses the pooling of spikes in multi units should not have severely effected the response pattern in most cases.

### Parcellation of the AC in *P. discolor*

One way of defining different fields of AC is by comparing the afferent connections with the auditory thalamus. Common to all mammals is a strong tonotopically organized projection from the ventral division of the medial geniculate body (MGBv) to AI [45]. In contrast to this, non-tonotopically organized non-primary cortical areas receive major projections from the dorsal division of the MGB (MGBd) [45]. CF/FM bats like horseshoe bats (e.g. *R. rouxi*, [39,41]) and *P. parnellii *[46] follow this connectivity pattern. In both animals, AI contains a tonotopic map where frequencies of the CF-components of the calls are overrepresented while frequencies of the FM-components are largely absent [18]. In *R. rouxi*, neurons specialized for the processing of acoustic parameters relevant for echolocation (e.g. echo delay) are mainly represented in dorsally located cortical areas that are targeted by projections from the MGBd [41]. The view of a congeneric projection from MGBv to AI covering the entire frequency range has been challenged by recent findings in the gleaning bat *Antrozous pallidus*. In this bat, the low frequency portion of a coherent frequency representation in the AC is innervated by the MGBv, whereas the high-frequency part received major projections from the supra-geniculate nucleus, a part of MGBd [47]. Neurons in the high-frequency range were moreover of a distinct binaural input type (EO) and preferred FM-sweeps, whereas neurons in the low frequency part were classified as EI and responded preferentially to noise. Two functionally distinct pathways evidently exist in this bat species: First, a low frequency pathway serving for passive prey location and second, a high-frequency pathway serving for obstacle avoidance during active echolocation [47,48]. The approach that part of AI may not receive input from the MGBv challenges one of the traditional definitions for AI via connectivity that, however, does not constitute the only valid criterion.

The distribution of binaural response types in *P. discolor *resembles that in *A. pallidus*, whereas neuroarchitectonic and neurophysiological features argue for a cortical fields pattern of the AC in *P. discolor *similar to the arrangement in *R. rou*xi and other mammals. Tracer experiments were not included in the present study in *P. discolor*, and consequently data on thalamo-cortical connections are not available. However, neuroarchitectonic features provide valuable tools for the definition of subdivisions in AC when combined with neurophysiological outcomes. Undoubtedly, the combination of all three approaches would yield optimal possibilities to classify cortical subdivisions. The specific usefulness of different neurohistological stains to determine cortical fields will be discussed in the following: The staining for zinc proved to be specifically helpful, complementing staining for cells and myelinated fibers. The zinc stain reveals horizontal bands in the neocortex that show characteristic variation in width and staining intensity among cortical regions, sharply defining the borders between cortical areas and their subdivisions, and are generally coincident with the limits defined in the Nissl stained sections. The banding patterns are stable among the species studied so far, allowing for an interspecies comparison (e.g. rats: [49-51] ; mice: [52] ; cats: [53] ; wallabies: [54] and humans [55]).

The PVF shows several features in the zinc stain characterizing primary sensory regions in the mammalian neocortex: conspicuous pale zinc staining of the outer portion of layer I, the differentiation of layer V in sublayers and, most characteristic, a wide, light band corresponding to layer IV encompassing the lower portion of layer III. In PDF, most distinctive to the PVF is the zinc staining of layer IV, which is narrower and more heavily stained, whereas layer V is thicker and of higher staining intensity. Zinc staining in AVF is generally intense. These features of PDF and ADF have been described to be characteristic for secondary sensory regions (rats: [49-51] ; mice: [52] ; cats: [53] ; wallabies: [54] and humans [55]). Furthermore, the structure of ADF seems unique in that it could be an amalgam of auditory and neighboring non-auditory, probably somatosensory areas. Mixed auditory-somatosensory fields neighboring the AC rostrally have been reported for several mammalian species (flying fox and gray squirrel: [56,57] ; mouse: [58] ; cat: [59] ; rat: [60]).

The accordance of response properties of cortical units in AVF and PVF with the anatomical partitioning of ventral auditory fields in the cortex is a further strong support, that the PVF might correspond to AI in *P. discolor*. PVF is tonotopically organized with BFs increasing from caudal to rostral. The AVF shows also a tonotopic organization but in contrast to the PVF, BFs increase from rostro-ventral to caudo-dorsal locations. This organization is in accordance to findings in other mammals where tonotopic gradients with roughly opposite directions have also been identified in the AI and the AAF (e.g. cat: [61]; ferret: [62]; gerbil: [63]; bat: *C. perspicillata *[20]). Because of the common high-frequency border of the AVF and PVF, high frequencies are represented all the way along the medial border of the PVF down to the most ventral border of this field (see Fig. 6A). In addition, no clear tonotopic gradient can be seen in the PDF that could be a tonotopic continuation of the frequency representation in the ventrally located PVF. Thus, it is unlikely that the PDF just represents the high-frequency portion of a contiguous frequency representation starting with low frequencies in the PVF as assumed for the AC of *A. pallidus *[47]. It is more likely that the PDF represents a non-tonotopically organized field like the dorsal fields found in other bats before (e.g. *R. rouxi *[13] and *C. perspicillata *[20], see above). However, also in *C. perspicillata *information about the thalamo-cortical connections is lacking.

In addition to BF, the distribution of several response properties like duration and first spike latency showed significant differences between the PDF and the PVF. To further test the possibility that high-frequency units in the PDF and PVF belong to a contiguous frequency representation like in *A. pallidus *[47], we additionally tested the differences of latency and response duration only for the high-frequency units (BF > 50 kHz) of both cortical fields. Latency and response duration for high-frequency units alone were still significantly different (Mann-Whitney U test, p < 0.05) between PDF and PVF. These findings further strengthen our parcellation of the AC based on the anatomical findings.

## Conclusion

In this study, four subfields were identified in the AC of the bat *P. discolor*. These differed in their neuroanatomical attributes and in the response properties of their units to pure tone stimulation. The neuroanatomical and neurophysiological properties of the tonotopically organized PVF reflected common characteristics of AI in the mammalian AC (e.g. very wide and pale zinc staining band in layer III/IV, BF gradient ascending from caudal to rostral). The AVF located rostrally to the PVF showed a tonotopic BF gradient running roughly in opposite direction to the gradient of the PVF. Thus, the AVF of *P. discolor *might represent the AAF described in other mammals. The dorsal part of the AC of *P. discolor *was non-tonotopically organized with BFs mainly in the high-frequency range. As most energy of the echolocation pulse of this bat species is contained in the frequency range between 60 to 80 kHz, the dorsal region of the AC of *P. discolor *seems to be particularly important for echolocation. With regard to the relative position of cortical fields and their basic properties of BF representation, the AC of *P. discolor *seems to follow the organizational pattern seen in other phyllostomid bats.

To further compare the parcellation of the AC of *P. discolor *with those of other bats and mammals, the specific connectivity between subdivisions of the AC and different divisions of the auditory thalamus of *P. discolor *must be investigated in future studies.

## Methods

### Experimental animals

All experiments were performed in agreement with the principles of laboratory animal care and under the regulations of the current version of German Law on Animal Protection (approval 209.1/211-2531-68/03 Reg. Oberbayern).

Five male and five female adult spear-nosed bats (*Phyllostomus discolor*, body weight: 30 to 45 g) were used in this study. The animals originated from a breeding colony in the Department Biology II of the Ludwig-Maximilians-University in Munich. For experiments, animals were kept separated from other bats under semi-natural conditions (12 h day/12 h night cycle, 65 to 70 % relative humidity, 28°C) with free access to food and water.

### Anesthesia and surgical preparation

In a pre-recording surgical session, the bat was prepared under anesthesia to provide for stable stereotaxic fixation in well defined coordinates and proper access to the target area. For anesthesia, a combination of Medetomidin (Domitor^®^, Novartis, Mississauga, Canada), Midazolam (Dormicum^®^, Hoffmann-La Roche, Mississauga, Canada) and Fentanyl (Fentanyl-Janssen^®^, Janssen-Cilag, Neuss, Germany) was injected subcutaneously (MMF, 0.4, 4.0 and 0.04 μg/g body weight). Skin and muscles covering the upper part of the cranium were cut rostro-caudally along the midline and shifted aside laterally. The cranial bone was completely cleaned of all remaining tissue and a small metal rod was fixed onto the caudal part of the bat's skull using light-curing dental cement (Charisma^®^, Heraeus Kulzer, Wehrheim Germany). After surgery, the anesthesia of the animal was antagonized by injecting a weight-dependent dose of a mixture of atipamezole hydrochloride (Antisedan^®^, Novartis), Flumazenil (Anexate^®^, Hoffmann-La Roche) and Naloxon (DeltaSelect^®^, DeltaSelect, Dreieich, Germany) subcutaneously (AFN: 8.1, 0.034 and 0.32 μg/g bodyweight). To alleviate postoperative pain, the analgesic Meloxicam (Metacam^®^, Boehringer-Ingelheim, Ingelheim, Germany, 0.2 mg/kg bodyweight) was administered after full recovery of the bat.

### Stereotaxic procedure and verification of recording sites and correlation with neuroarchitectural features

A detailed description of the stereotaxic procedure, determination of brain orientation and reconstruction of recording sites has been already published elsewhere [64]. In brief, the animal's head was fastened with the surgically mounted rod to a stereotaxic frame in a well defined orientation. The alignment of the animal's skull and consequently the brain within the stereotaxic coordinate system was measured by scanning characteristic profile lines of the skull. The profile lines were measured in parasagittal and frontal planes (500 μm steps) relative to a fixed reference point and compared to a profile defining a standardized orientation of the skull and brain of *P. discolor *(Fenzl and Nixdorf, unpublished data), that also determines the normal axes of the available brain atlas of the animal. Thus, deviations of the actual alignment of the bat's head from the standardized position could be corrected by appropriate tilt and pitch of the animal within the experimental setup. Any reorientation of the skull is thus under accurate stereotaxic control. This procedure allowed the pooling and comparison of all electrophysiologically measured data, brain lesions and tracer deposits within one and among different experimental animals. Locations of lesions or marker (HRP, WGA-HRP) deposits were determined after transcardial perfusion and subsequent histological processing of the brain based on the brain atlas. These atlas-oriented coordinates were compared with those of the physiologically determined reconstruction of the specific location. This allowed a verification of the validity of recording coordinates, or eventually indicated errors that could typically be corrected after retracing the mistake in localization.

For the analysis of the neuroarchitectural features of the auditory cortex, series of frontal sections stained for cresyl violet, myelin [65] and zinc were available. For the detection of zinc, brains were processed according to a modification of the Timm method [66]. Zinc plays an important role in cell physiology [67] and has synaptic signaling functions in the mammalian brain [68,69]. In the neocortex, the stain for zinc reveals horizontal bands that show marked variation in width and staining intensity among functionally different cortical regions. These characteristic banding patterns are generally coincident with the limits defined in the Nissl stained sections, but have the advantage that they are readily noticeable at low magnification.

Additional series of frontal sections stained for the calcium-binding proteins, acetylcholine-esterase (ACHe), cytochrome-oxidase were not included in the analysis presented here, as they did not allow the differentiation between core and belt regions as clearly as described for other mammals with a more highly evolved neocortex (e.g., ACHe: [70,71]; Cytochromoxidase: [72]; calcium-binding proteins: [73,74]). Therefore, although involving a complicated perfusion procedure, the stain for zinc was chosen as histochemical marker to delineate cortical areas.

### Acoustic stimuli and recording of neuronal responses

All experiments were conducted in a heated (36°C), electrically shielded and anechoic chamber. They normally lasted 4 hours per session and were performed at four days a week for up to six weeks. For each session, the bat was anaesthetized using MMF (see above). Throughout the whole experiment the animals were supplied with oxygen. To lower electrodes into the brain areas for recording of neural responses, small holes of about 500 microns in diameter were drilled into the animal's skull above the auditory cortex and the dura was perforated. Electrode penetrations were generally carried out in tangential direction, roughly parallel to the surface of the brain (bat #1–5, #8 and #9) or in a roughly perpendicular direction to the brain surface (bat #10). Two mapping experiments were done with orthogonal electrode penetrations (bat #6 and #7) to cover the entire auditory cortex in single individual bats and to neurophysiologically outline the borders and reveal possible tonotopic trends of the AC as a whole. Only basic properties like tuning and threshold were recorded in these two experiments.

Search stimuli for neuronal activity were pure tones with 20 ms duration produced with a frequency generator (Wavetek model 136, 186, FG-5000) and a custom-made pulse former. The attenuation of these stimuli could be modified manually (external attenuator AP401, adret electronique, France). The stimuli were presented via custom-made ultrasonic earphones [75] with a flat frequency response (± 3 dB SPL between 10 to 100 kHz). As it was not always possible to discriminate the activity of a single neuron, the term 'unit' is used in the text to describe the activity of one neuron to clusters of up to three neurons recorded at a distinct recording site. Once a unit was detected, its BF was determined audio-visually. For most units, the frequency response area (FRA) was determined in more detail. Therefore, pure tone stimuli (20 ms duration, 2 ms rise and fall time), in various frequency and sound pressure level combinations were presented contralaterally. Although *P. discolor *emits FM-pulses for echolocation, pure tone stimuli were used to allow comparison of the data to a previous study in a closely related phyllostomid bat, *C. perspicillata*, [20]. The stimulus duration of 20 ms is roughly intermediate between the short echolocation pulses (< 3 ms) and longer social calls (about 50 ms, [76]) of *P. discolor*.

The binaural response properties were determined at the unit's BF by using ABI (averaged binaural intensity, [77]). 20 ms long pure tones were presented binaurally with increasing intensity at contralateral and decreasing intensity at ipsilateral side and vice versa. The interaural intensity difference (IID) was changed from -20 dB SPL to +20 dB SPL in steps of 5 dB SPL. The level of the signals was chosen so, that both ears were stimulated at 20 dB SPL above the unit's auditory threshold. Thereafter, the same stimuli were presented monaurally to the contralateral and ipsilateral ear.

All stimuli for neuronal recordings were computer-generated (Matlab^® ^6.1; Mathworks, Natick, USA), digital-analog converted (RX6; sampling rate 260 kHz, Tucker Davis Technologies, Gainsville, USA) and attenuated (PA5, Tucker Davis Technologies, Gainsville, USA). The acoustical stimulus parameters (frequency/sound pressure level combinations) were presented pseudo-randomly with a repetition rate of 1.3 Hz, a silent interval of 10 to 50 ms leading stimulus onset and with 10 repetitions of the entire parameter set.

Responses from units in the auditory cortex were recorded extracellularly by using either borosilicate glass electrodes (#1B100F-3, WPI, Sarasota, USA) filled with 2 M NaCl and 4 % pontamine sky blue (3 to 8 MΩ impedance), carbon fiber microelectrodes (Carbostar-1, Kation Scientific, Minneapolis, USA; 0.4 to 0.8 MΩ impedance) or glass insulated tungsten microelectrodes (Alpha Omega GmbH, Ubstadt-Weiher, Germany, 1 to 2 MΩ impedance). Responses were amplified (Electro 705, WPI; ExAmp-20 FB, Kation Scientific or RA16PA, Tucker Davis Technologies, Gainsville, USA for glass microelectrodes, carbon fiber electrodes or tungsten microelectrodes, respectively), band-pass filtered and fed into an A/D-converter (RP2.1 or RX5, Tucker Davis Technologies, Gainsville, USA, sampling rate: 25 kHz). Using the stimulation and analysis software "Brainware" (J. Schnupp, distributed by Tucker Davis Technologies, Gainsville, USA) action potentials were threshold discriminated and saved for offline analysis on a personal computer.

### Data analysis

All non-commercial computer programs used for data analysis were written in Matlab^®^. Data analysis was done based on peri-stimulus time histograms (PSTH, 1 ms bin width) constructed from the spike responses to different parameter sets. The time window for analysis was adjusted to the total response duration of the unit. It started when the first bin exceeded the level of spontaneous activity and ended when the response reached steady spontaneous level again. The level of spontaneous activity was derived from the silent period preceding each stimulus onset. First spike latency was determined for stimuli presented at the unit's BF at a level of 20 dB SPL above threshold (see below for details on BF and threshold). The latency of the first spike to each presentation was used to calculate the median value of first spike latency. Only spikes occurring within the time window for analysis were included in the calculation.

The response characteristics of units that were tested at different combinations of frequency and sound pressure level, were visualized as FRA constructed from the summed activity within the given time window. Responses at different frequency level combinations were considered to be significant if the spike rate exceeded 20 % of the absolute maximum response of the unit. Basic characteristic values of the units like best frequency and tuning quality (Q_10dB _and Q_30dB_) were directly derived from the FRA. The data basis for best frequency thresholds is the same as used for a previous publication [78]. However, data was re-analyzed in the present paper regarding the topographical distribution of thresholds within the AC of *P. discolor*. FRAs of units were classified as V-shaped, circumscribed, double-tuned or complex following roughly the categories used by Heil and Irvine [79] and Sutter [80]. The spike rate of units with circumscribed FRAs decreased at all frequencies to zero with increasing stimulus level. Double tuned units had FRAs that showed two clearly separable response regions, whereas complex FRAs consisted of multiple activity patches separated by regions of low activity. Cortical units classified as V-shaped responded over a contiguous range of frequency level combinations. V-shaped and double tuned FRAs were further distinguished according to their spike rate level function as monotonic or non-monotonic. The unit was labeled as non-monotonic if the spike-rate decreased again to below 75 % of the maximum at BF at higher levels. Thus, units with circumscribed FRAs always featured non-monotonic spike-rate-level functions by definition. Q_10dB _values as a measure of sharpness of frequency tuning were calculated using the following equation: Q_10dB _= BF/bandwidth measured 10 dB SPL above threshold. Double tuned and complex units did not generally allow determining a Q_10dB _value. If the shape of FRAs allowed, Q_30dB _values were also measured.

To determine a unit's binaural properties, the mean number of spikes was plotted as a function of IID for binaural, monaural contralateral and monaural ipsilateral stimulation. Comparison of these curves allowed the grouping of the binaural properties into four categories. Units that were excited by monaural stimulation of each ear were named EE (excitatory-excitatory). Units that only received excitatory input from the contralateral ear are named E0 (excitatory input only from the contralateral ear). In case the unit was excited by monaural contralateral stimulation, did not respond to monaural ipsilateral stimulation and showed inhibition in binaural stimulation, it was classified as EI (excitatory-inhibitory). The last group ("other") contained units that could not be ranked into one of the previous groups.

The locations of recording sites were projected to the flattened surface of the auditory cortex. For a detailed description of the surface projection method, see [81]. Briefly, each recording site was projected to the location at the cortical surface that had the shortest distance to the recording site. For the flattening process, the distance of the projected recording site to the origin fixed at 2000 μm lateral from the midline of the brain (upper blue line in Fig. 1B) was calculated. Thus, distortions due to irregularity of the cortical surface (e.g. pseudocentral sulcus) were avoided.

Distribution and trends of parameters on the cortical surface are represented with the help of the Voronoi tessellation procedure in two dimensions implemented with Matlab^®^. In detail, recording sites of the chosen parameter are connected with all neighboring recording sites. Cells characterizing the value of a special parameter around recording sites are constructed as polygons whose sides pass equidistantly between recording sites and cross the connection lines perpendicularly.

## Authors' contributions

SH, UF, GS and BS performed the electrophysiological experiments. SR–S performed the histological and neuroanatomical procedures. SH, UF and SR–S analyzed the data. SH, UF, SR–S and GS wrote the paper.
